# Application of autonomic nervous function evaluation to job stress screening

**DOI:** 10.1016/j.heliyon.2019.e01194

**Published:** 2019-02-05

**Authors:** Naoko Okawa, Daisuke Kuratsune, Junichi Koizumi, Kei Mizuno, Yosky Kataoka, Hirohiko Kuratsune

**Affiliations:** a Department of Health Welfare, Kansai University of Welfare Sciences, Osaka, Japan; b Fatigue Science Laboratory Inc., Osaka, Japan; c Department of Metabolism, Endocrinology, and Molecular Medicine, Osaka City University Graduate School of Medicine, Osaka, Japan; d Yokohama National University, Kanagawa, Japan; e The Open University of Japan, Japan; f RIKEN Center for Biosystems Dynamics Research, Hyogo, Japan; g RIKEN Compass to Healthy Life Research Complex Program, Hyogo, Japan; h Department of Medical Science on Fatigue, Osaka City University Graduate School of Medicine, Osaka, Japan; i Kokorotics Inc., Hyogo, Japan

**Keywords:** Physiology

## Abstract

The present study focuses on the evaluation of autonomic nervous function, which is increasingly being used as an objective measure of fatigue state. It has recently been reported that autonomic nervous activity, which is expressed as total heart rate variability (HRV) power, is associated with, and can be used as an objective measure of, mental and physical fatigue. Total HRV power (log (LF + HF)) has been shown to decline with ageing, and thus cannot be utilized as a fatigue index in populations with a different age composition. In the present study, we devised standard scores (deviation value) for autonomic nervous activity corrected for individual age calculated from the distribution of such activity in individual age cohorts. This allowed us to accurately evaluate an individual's autonomic nervous activity, even when that individual was part of a group with members of different ages. Standard scores were quantified using autonomic nervous function data gathered from 1,969 healthy individuals (age range 20–77 years).

The efficacy of this method in mental health screening was investigated by evaluating both autonomic nervous function and subjective levels of fatigue among corporate workers. Based on results from the Brief Job Stress Questionnaire recommended by the research team of the Ministry of Health, Labour and Welfare, 103 participants were divided into two groups (a high-stress group [n = 17] and a non–high-stress group [n = 86]). Visual analog scale (VAS) scores for all fatigue-related symptoms were significantly higher among the high-stress than among the non–high-stress group (p < 0.01).

The mean standard score for autonomic nervous activity was 56.3 for the non–high-stress group. The score for the high-stress group was significantly lower, at 47.9 (p < 0.01), indicating that autonomic nervous function was reduced among participants who experienced high stress. According to an analysis of raw and standard scores in each domain, autonomic nervous activity did not significantly correlate with stress-causing factors (e.g., overwork) or other factors affecting stress responses (e.g., support from supervisors and colleagues), but did exhibit a significant positive correlation with physical and mental responses to stress (r = 0.334, p < 0.01). Lower raw scores for mental and physical responses to stress represent stronger subjective symptoms. Moreover, greater stress responses were found to be associated with lower standard scores for autonomic nervous activity.

In terms of fatigue-related symptoms rated using the VAS, autonomic nervous activity negatively correlated with mental stress, physical stress, fatigue/malaise, depressed mood, anxiety/fear, tension, irritation/anger, cognitive decline, and muscle/joint/general pain, and positively correlated with motivation/vitality. Reduced autonomic nervous activity was observed with high stress, confirming that standard scores for autonomic nervous activity are associated with mental and physical responses to stress and subjective fatigue-related symptoms.

These results indicate that the evaluation of autonomic nervous activity using standard scores (deviation value) is a useful tool for the objective measurement of fatigue state, even in groups with members of different ages, and can be applied as a useful objective health index to evaluate industrial fatigue.

## 1 Introduction

In recent years, Japan has seen an upward trend in the number of employees who have received approval for workers' compensation after developing psychiatric disorders owing to job stress. As such, a major priority for the country is to address the demand for primary prevention, that is, measures to preempt mental health impairment among workers. Fatigue is a natural biological response that can affect anyone who continuously carries out activities of daily living. However, if left unmanaged, fatigue becomes chronic, leading to a state of overwork and subsequently, an increased risk of developing disorders. In addition to personal health, worker fatigue is an issue that impacts both safety and productivity at the workplace as a whole; therefore, it is important for workers and corporations to thoroughly consider and implement countermeasures. Such workplace countermeasures are even more imperative in view of recent problems related to acute fatigue evolving into a chronic state [1].

A Basic Survey on Industrial Safety and Health conducted in 2012 by the Ministry of Health, Labour and Welfare (MHLW) found that the rate of respondents who experience strong anxiety, worry, and stress about their jobs and working life reached approximately 60%. The MHLW also discovered from a patient survey in 2014 that more than 1.11 million patients suffer from mood disorders such as depression, and this number has been rising annually. The significance of corporate mental health management began garnering attention as more workers took administrative leave owing to mental impairment, and the Japanese government responded to this situation by formulating a stress check program at the time the Industrial Safety and Health Act was revised in December 2015 [2, 3, 4]. This fatigue and stress check program mandates corporations to administer examinations performed by industrial physicians and public health nurses to assess employees' levels of psychological burden. The MHLW research team currently recommends the use of the Brief Job Stress Questionnaire (BJSQ) as a primary instrument for stress evaluation.

In addition to workers' health status (e.g., fatigue, depressed mood, anxiety, irritation), the BJSQ also measures aspects such as support in the workplace and qualitative and quantitative workload. In other words, if properly completed, the questionnaire can be useful for appraising not only the health of individuals, but also the working environment.

Nevertheless, industrial physicians and public health nurses practicing at workplaces have voiced concerns about the difficulties in reaching a proper diagnosis using the BJSQ, which is a subjective evaluation method. Since many workers who experience anxiety, depressed mood, and/or fatigue tend to mask their abnormalities in the BJSQ test, objective health assessment methods in combination with the BJSQ are desired.

In recent years, it has become clear that several evaluation methods can objectively judge fatigue state. One such method is autonomic function evaluation. Because self-regulation and autonomic regulation colocalize in the brain, an autonomic measure, heart rate variability (HRV), has been reported to provide an index of self-regulatory strength and activity [5]. Because self-regulation and autonomic regulation colocalize in the brain, HRV, an autonomic measure, has been reported to provide an index of self-regulatory strength and capacity and to modulate autonomic nervous activity. Segerstrom et al. [5] reported that higher resting HRV has been associated with a number of potential indicators of self-regulatory capacity, fewer negative emotions during daily stress, more effective stress coping, and better impulse control. They also suggested that self-regulatory tasks are associated with activation of the prefrontal cortex, and that self-regulatory fatigue selectively and adversely affects performance on cognitive tasks that are considered to be frontal or executive. When we studied brain function using H_2_O^15^ and positron emission tomography [6], we also found that the deterioration of prefrontal cortex function was associated with fatigue in patients with chronic fatigue syndrome.

It has also been reported that increased sympathetic and decreased parasympathetic activity may be characteristic features of both acute and daily levels of fatigue [7]. Sympathetic hyperactivity based on decreased parasympathetic activity has also been found to be associated with mental fatigue induced by prolonged cognitive load [8]. Furthermore, when we assessed autonomic function in a mental fatigue model of healthy volunteers caused by long-term computerized Kraepelin test workload, the low/high frequency component ratio (LF/HF) was significantly increased by the fatigue-inducing task and decreased by resting, suggesting that mental stress causes a relatively sympathetic nerve activity-dominant state [9]. When we studied autonomic nerve function in patients with chronic fatigue syndrome, we found that as the symptoms of fatigue became stronger, parasympathetic nervous activity decreased. As a result, the stronger the fatigue symptoms, the more the sympathetic nervous system becomes significant in autonomic nerve function [10].

However, when we studied autonomic nervous activity as expressed by total HRV power (log (LF + HF)) in 428 healthy persons, it was found to be associated with age, and because it has been observed to decline with ageing [11], it cannot be used as a fatigue index in populations with a different age composition.

## 2 Materials & methods

Between September 13, 2016 and February 27, 2017, a device that measures autonomic nervous activity was placed at the offices of 17 corporations in Kanagawa Prefecture. Data from 103 employees (52 men, 51 women; mean age, 39.1 ± 9.2 years) working in these offices were obtained through autonomic measurement based on analysis of HRV, measurement of fatigue-related symptoms with 10 items rated on a visual analog scale (VAS), and results from the BJSQ (57 questions) recommended by an MHLW research team. The present study was conducted as part of the “MY ME-BYO Record” Project in Kanagawa Prefecture (Japan).

### 2.1 Autonomic mesurement based on analysis of HRV

The evaluation of autonomic nervous function was performed by a method previously described [12]. Briefly, participants underwent simultaneous electrocardiography and photoplethysmography using a Vital Monitor 302 system (Fatigue Science Laboratory, Osaka, Japan) while sitting quietly with their eyes closed for 2 minutes. These data were analyzed using MemCalc software (GMS, Tokyo, Japan). Frequency analyses for R-R interval variation from electrocardiography and a-a interval variation as the second derivative of photoplethysmography (accelerated plethysmography) were performed using the maximum entropy method, which is capable of estimating the power spectrum density from short time series data and is adequate for examining changes in HRV under different conditions of short duration [13, 14]. The power spectrum resolution was 600 Hz. For frequency analyses, the LF component was calculated as the power within a frequency range of 0.04–0.15 Hz, and the HF component was calculated as the power within a frequency range of 0.15–0.4 Hz.

HF is vagally mediated [15, 16, 17], whereas LF originates from a variety of sympathetic and vagal mechanisms [12, 18]. Some review articles [19, 20, 21] have noted that LF reflects sympathetic nerve activity and has been used as a marker of sympathetic nerve activity. In the present study, a practice test was conducted for 1 minute before autonomic nerve function testing was conducted for 2 minutes, in accordance with previous studies [22, 23, 24]. The reliability of these tests has been confirmed [24, 25].

Autonomic nervous activity as expressed by total HRV power (log(LF + HF)) is associated with age and has been observed to decline with ageing, so it cannot be used as a fatigue index in populations with a different age composition. Therefore, in the present study, we devised standard scores (deviation value) for autonomic nervous activity corrected for individual age (Patent pending; Koizumi et al.) [11].

### 2.2 Measurement of fatigue-related symptoms with 10 items on a VAS

This method was used to determine current subjective levels of fatigue. Rated items included mental stress, physical stress, fatigue/malaise, depressed mood, motivation/vitality, anxiety/fear, tension, irritation/anger, cognitive decline, and muscle/joint/general pain. All items were rated on a scale ranging from “none at all” to “worse than ever before”.

### 2.3 MHLW-recommended BJSQ comprising 57 questions [26, 27, 28]

The MHLW-recommended BJSQ was created to analyze current sources and levels of stress among respondents. The simple structure and modest number of items allows for user-friendly on-site measurement and evaluation. In addition to being compatible with a wide range of workplaces, the BJSQ is known to exhibit a high level of reliability and validity in identifying both positive and negative psychological stress responses. Results of the 57-question BJSQ are divided into three domains for evaluation: factors believed to cause stress, such as qualitative and quantitative workload (Domain A); mental and physical responses triggered by stress, such as fatigue, depressed mood, anxiety, and irritation (Domain B); and other factors affecting stress responses, such as levels of support from supervisors, colleagues, and family members (Domain C).

Results are tallied with a raw score conversion table, with low scores representing higher levels of stress. A state of high stress is indicated when increases in subjective fatigue-related symptoms lead to a total Domain B score of ≤12, and when insufficient social support and greater qualitative and quantitative workload lead to a sum of Domain A plus Domain C scores of ≤26, even if the Domain B score is ≤17.

### 2.4 Statistical analysis

Regarding statistical evaluation, the Mann-Whitney U test was conducted to compare each item in the evaluation of subjective fatigue. For the items in the evaluation of autonomic nervous function, a two-sample t test was performed by logarithmizing the calculated values, with a significance level of 5%.

### 2.5 Ethical treatment

All experiments were conducted in compliance with national legislation and the Code of Ethical Principles for Medical Research Involving Human Subjects of the World Medical Association (Declaration of Helsinki). The study protocol was approved by the Ethics Committee of Kansai University of Welfare Sciences (Approval number: 14-25), and all participants provided written informed consent for participation in the study.

## 3 Results

### 3.1 Evaluation of stress state using the Brief Job Stress Questionnaire (BJSQ)

**Fig. 1:**
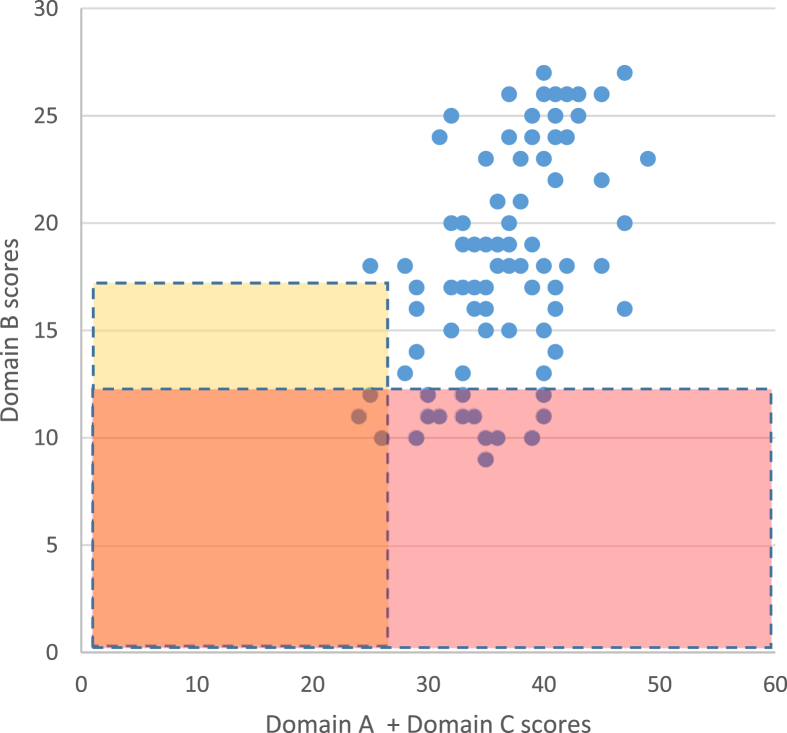
Results from the Brief Job Stress Questionnaire (BJSQ). Results from the 57-question BJSQ were divided into three domains for evaluation: factors believed to cause stress, such as qualitative and quantitative workload (Domain A); mental and physical responses triggered by stress, such as fatigue, depressed mood, anxiety and irritation (Domain B); and other factors affecting stress responses, such as levels of support from supervisors, colleagues, and family members (Domain C).

BJSQ scores demonstrated that 17 (16.5%) of the 103 participants were in a state of high stress (Fig. 1).

### 3.2 Evaluation of domains A, B, and C in the high-stress group and the non–high-stress group

Table 1 shows the results of Domains A, B, and C in the high-stress group and the non–high-stress group. Results were tallied using a raw score conversion table, with low scores representing higher levels of stress. Scores were significantly lower for the high-stress group in Domains A (24.5 ± 3.8), B (10.9 ± 0.9), and C (7.8 ± 2.6) than those for the non–high-stress group in Domains A (27.9 ± 3.0) (p < 0.01), B (19.9 ± 4.1) (p < 0.001), and C (9.6 ± 2.8) (p < 0.05). The results in Domain A mean that the high-stress group had a high amount of work, a high degree of demand, and low job control compared with the non–high-stress group, the results in Domain B mean that the high-stress group had higher symptoms (fatigue, depression, and anxiety) in the fatigue state, and the results in Domain C mean that the high-stress group had a lower level of support from supervisors, colleagues, and family members.

**Table 1:** Relationship between Domain A, B, and C scores and stress groups.

	Domain A scores	Domain B scores	Domain C scores
high-stress group (n = 17)	24.5 ± 3.8	10.9 ± 0.9	7.8 ± 2.6
non–high-stress group (n = 86)	27.9 ± 3.0	19.9 ± 4.1	9.6 ± 2.8
p-value	p < 0.01	p < 0.001	p < 0.05

### 3.3 Relationship between stress and fatigue-related symptoms rated using a VAS

**Fig. 2:**
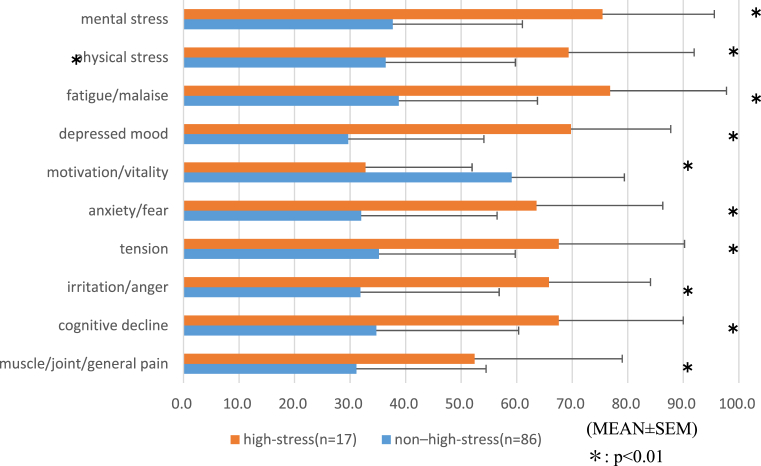
Fatigue-related symptoms among the high- and non–high-stress groups measured using a visual analog scale (VAS).

Based on the results of the BJSQ, the 103 participants were divided into two groups (a high-stress group [n = 17] and a non–high-stress group [n = 86]). VAS scores for all fatigue-related symptoms were significantly higher among the high-stress than among the non–high-stress group (p < 0.01) (Fig. 2).

### 3.4 Relationship between stress and autonomic nervous function

The total HRV power (log (LF + HF)) for autonomic nervous activity was 2.9 ± 0.5 in the non–high-stress group (n = 86) and 3.1 ± 0.5 in the high-stress group (n = 17). No significant difference was observed between the two groups (Table 2). However, the standard scores for autonomic nervous activity were 56.3 ± 10.6 for the non–high-stress group (n = 86) and 47.9 ± 10.7 for the high-stress group (n = 17), representing a significant difference between the two groups (p < 0.01) (Table 2). Since autonomic nervous activity as expressed by total HRV power (log (LF + HF)) is associated with age, it cannot be used as a fatigue index in populations with a different age composition.

**Table 2:** Relationship between autonomic function and stress groups.

	logTP(LF + HF)	ANA standard scores∗	log(LF/HF)
high-stress group (n = 17)	3.1 ± 0.4	47.9 ± 10.7	0.0 ± 0.3
non–high-stress group (n = 86)	2.9 ± 0.5	56.3 ± 10.6	0.1 ± 0.4
p-value	ns	p < 0.01	ns

∗ standard scores for autonomic nervous activity as expressed by total HRV power (log(LF + HF)).

The autonomic nerve balances (log (LF/HF)) were 0.1 ± 0.4 in the non–high-stress group (n = 86) and 0.0 ± 0.3 in the high-stress group (n = 17). However, no significant differences were observed between the two groups (Table 2).

### 3.5 Relationship between raw scores for each domain and standard scores for autonomic nervous activity

**Fig. 3:**
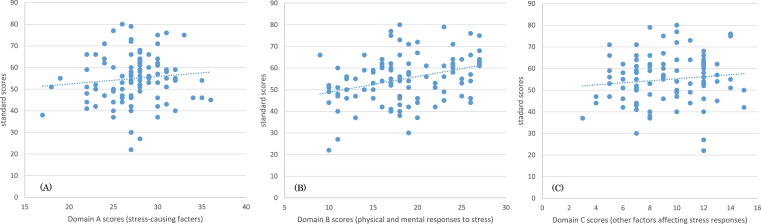
Relationship between standard scores for autonomic nervous activity and domain scores (A, B, and C). A (left): Relationship with factors believed to cause stress, such as qualitative and quantitative workload (Domain A); B (center): Relationship with mental and physical responses triggered by stress, such as fatigue, depressed mood, anxiety, and irritation (Domain B) (r = 0.334, p < 0.01); C (right): Relationship with other factors affecting stress responses, such as levels of support from supervisors, colleagues, and family members (Domain C).

According to an analysis of raw and standard scores in each domain, autonomic nervous activity did not significantly correlate with stress-causing factors (e.g., overwork) (Fig. 3A) or other factors affecting stress responses (e.g., support from supervisors and colleagues) (Fig. 3C), but did exhibit a significant positive correlation with physical and mental responses to stress (Fig. 3B) (r = 0.334, p < 0.01). Lower raw scores for mental and physical responses to stress represent stronger subjective symptoms. Moreover, greater stress responses were found to be associated with lower standard scores for autonomic nervous activity.

### 3.6 Relationship between autonomic nervous activity and fatigue-related symptoms rated using a VAS

In terms of fatigue-related symptoms rated using the VAS, autonomic nervous activity negatively correlated with mental stress, physical stress, fatigue/malaise, depressed mood, anxiety/fear, tension, irritation/anger, cognitive decline, and muscle/joint/general pain, and positively correlated with motivation/vitality (Table 3).

**Table 3:** Relationship between subjective symptoms measured using a visual analog scale and autonomic nervous activity.

		mental stress	physical stress	fatigue/malaise	depressed mood	Motivation/vitality	anxiety/fear	tension	irritation/anger	cognitive decline	muscle/joint/general pain
ANA standard scores∗	r	-0.2081	-0.2747	-0.2607	-0.2479	0.2641	-0.2459	-0.2527	-0.3221	-0.2857	-0.2933
	p	0.035	0.005	0.008	0.012	0.007	0.012	0.010	0.001	0.003	0.003

∗ standard scores for autonomic nervous activity as expressed by total HRV power (log(LF + HF)).

Reduced autonomic nervous activity was observed with high stress, confirming that standard scores for autonomic nervous activity are associated with mental and physical responses to stress and subjective fatigue-related symptoms (Table 1).

## 4 Discussion

Recently, more workers have been experiencing higher levels of stress in their jobs and at their workplaces, and suicide owing to such stress has become a major problem in society. Against this backdrop, in August 2000, the MHLW issued a brief titled “Guidelines for the Mental Health of Workers in the Workplace”. This brief advises businesses to prepare mental fitness plans and pursue four types of care activities: self-care that enables each and every worker to assess their own health status; line care, where every department in a corporation assesses the health status of their members; professional care, where industrial health specialists hired by the corporation provide aid; and outside care, where support can be sought from resources outside the corporation. The brief states that these activities should be implemented in a planned, continuous, and closely coordinated manner.

Therefore, as it becomes more important to promote self-care among workers, the adoption of a device that measures autonomic nervous function at the offices of corporations such as the one used in the present study is believed to help workers manage their own mental health and engage in employment with a stable frame of mind by giving them the means to objectively determine their state of fatigue. Another effective means of support could be in-house workshops that teach workers how to recognize stress and fatigue while sharing knowledge and practices that can be used as coping strategies on a regular basis.

The revision of the Industrial Safety and Health Act in June 2014 mandates businesses with 50 or more employees to conduct stress checks on all workers once a year [2, 3, 4]. Aiming to specifically reinforce primary prevention at the stage where mental health impairment can be preempted, the main purpose of the program is to “reduce stress among individual workers by regularly conducting tests to determine the status of worker stress and notifying workers of the results to promote self-recognition of their situation in regard to stress.”

Many corporations currently utilize the 57-question BJSQ developed by the MHLW research team. In addition to questionnaires, interviews are also an effective screening method for the early detection of individuals with potential mental health issues. However, interviews are only possible at corporations that can afford to hire experienced interview experts and can only be performed on a limited number of employees [29, 30]. Furthermore, because many workers with mental health issues deny having any problems in health status assessments with questionnaires, it is necessary to adopt an objective measure, something akin to a blood pressure monitor for mental health, to augment the BJSQ for preempting mental impairment.

The results of recent studies revealing sympathetic hypertonia and reduced autonomic nervous activity among teachers in a state of chronic fatigue and workers experiencing stress imply that evaluation of autonomic nervous function may be an effective means of measuring objective fatigue. However, age greatly influences autonomic nervous activity among individuals, making it difficult to apply this method to groups with members of different ages [9, 10, 30, 31].

The present study proposes a new indicator developed by employing autonomic nervous function analysis data from 1,969 healthy individuals to quantify autonomic nervous activity for every age cohort as standard scores for autonomic nervous activity. The relationship between measurements of autonomic nervous activity using this indicator, the results of the BJSQ, and levels of subjective fatigue-related symptoms were investigated in a group of 103 participants of various ages employed by 17 corporations across Kanagawa Prefecture.

Consequently, it was found that standard scores for autonomic nervous activity were significantly lower for participants assigned to a high-stress group according to their BJSQ results. No significant difference was found in autonomic nervous activity as expressed by total HRV power (log(LF + HF)) between the two groups (Table 2). As mentioned above, autonomic nervous activity as expressed by total HRV power (log(LF + HF)) is associated with age, so it cannot be used as a fatigue index in a group with different age composition.

This outcome suggests that objective fatigue evaluation with standard scores for autonomic nervous activity may be useful for analyzing fatigue among groups with members of different ages. With respect to fatigue-related symptoms, standard scores for autonomic nervous activity significantly negatively correlated with mental stress, physical stress, fatigue/malaise, depressed mood, anxiety/fear, tension, irritation/anger, cognitive decline, and muscle/joint/general pain, and positively correlated with motivation/vitality. This finding indicates that standard scores can be effective as an objective indicator for expressing pathological conditions of fatigue among individuals. To our knowledge, this is the first report to demonstrate a relationship between autonomic function and fatigue state in populations with a different age composition.

In light of past research demonstrating that individuals assigned to a high-stress group exhibited significantly higher rates of administrative leave, it is possible that objective fatigue evaluation with autonomic nervous function analysis can be useful for supplementing subjective health status surveys from the standpoint of preventing illness among workers [32].

Considering that the participants in the present study were employees of 17 corporations based in Kanagawa Prefecture, it is conceivable that the results may have been influenced by the specific characteristics of the region and cooperating businesses. Therefore, moving forward, it will be necessary to compare the findings of the present study with data obtained from workers in different regions and corporations.

## Declarations

### Author contribution statement

Naoko Okawa: Conceived and designed the experiments; Performed the experiments; Analyzed and interpreted the data; Wrote the paper.

Daisuke Kuratsune, Junichi Koizumi, Kei Mizuno, Yosky Kataoka: Performed the experiments; Analyzed and interpreted the data.

Hirohiko Kuratsune: Conceived and designed the experiments; Performed the experiments; Analyzed and interpreted the data; Wrote the paper.

### Funding statement

This research did not receive any specific grant from funding agencies in the public, commercial, or not-for-profit sectors.

### Competing interest statement

The authors declare no conflict of interest.

### Additional information

No additional information is available for this paper.
